# ﻿Four new species of *Pseudopoda* Jäger, 2000 (Araneae, Sparassidae, Heteropodinae) from China and Vietnam

**DOI:** 10.3897/zookeys.1230.142418

**Published:** 2025-03-06

**Authors:** He Zhang, Hailun Chen, Fan Zhang, Jie Liu, Peter Jäger, Qiangyong Fan, Lin Cheng, Changhao Hu

**Affiliations:** 1 Guo Shoujing Innovation College, Xingtai University, Xingtai 054001, Hebei, China Hubei University Wuhan China; 2 Hebei Province Sweet Potato Breeding and Application Technology Innovation Center, Xingtai 054001, Hebei, China Xingtai University Xingtai China; 3 Hubei Key Laboratory of Regional Development and Environmental Response, Faculty of Resources and Environmental Science, Hubei University, Wuhan 430062, Hubei, China Hebei Province Sweet Potato Breeding and Application Technology Innovation Center Xingtai China; 4 Hubei Key Laboratory of Resource Utilization and Quality Control of Characteristic Crops, College of Life Science and Technology, Hubei Engineering University, Xiaogan 432000, Hubei, China Hubei Engineering University Xiaogan China; 5 Arachnology, Senckenberg Research Institute, Mertonstraße 17–21, 60325 Frankfurt am Main, Germany Arachnology, Senckenberg Research Institute Frankfurt am Main Germany; 6 Jiangxi Wuyi Mountain National Nature Reserve Administration, Shangrao 334599, Jiangxi, China Jiangxi Wuyi Mountain National Nature Reserve Administration Shangrao China; 7 Hubei Broad Nature Technology Service Co. Ltd, Wuhan 430079, Hubei, China Hubei Broad Nature Technology Service Co. Ltd Wuhan China

**Keywords:** Asia, biodiversity, huntsman spiders, morphology, taxonomy

## Abstract

Four new species of *Pseudopoda* Jäger, 2000 (Araneae, Sparassidae) are described based on material collected from China and Vietnam: *P.campylotropa* Zhang, Chen, Liu, Jäger & Hu, **sp. nov.** (♂♀) and *P.caoguii* Zhang, Chen, Liu, Jäger & Hu, **sp. nov.** (♀) from Yunnan Province of China; *P.yejiachangensis* Zhang, Chen, Liu, Jäger & Hu, **sp. nov.** (♂) from Jiangxi Province of China; and *P.ornithorhynchus* Zhang, Chen, Liu, Jäger & Hu, **sp. nov.** (♂) from Vinh Phuc Province of Vietnam.

## ﻿Introduction

The spider genus *Pseudopoda* Jäger, 2000 is the largest genus within the family Sparassidae Bertkau, 1872, occurring in eastern, southern and southeastern Asia (Jäger et al. 2015) and currently comprising 264 species (World Spider Catalog 2025). Among them, 163 species are known in China, primarily distributed in southwestern regions, while only eight species have been recorded in Vietnam, primarily in northern areas (Jäger 2001; Jäger and Vedel 2005, 2007; Yang et al. 2009; Zhang et al. 2013a, 2013b; Logunov and Jäger 2015; Zhang et al. 2017; Jiang et al. 2018; Zhang et al. 2019; Yang et al. 2022; Zhang et al. 2023; Zhang et al. 2025).

*Pseudopoda* species primarily inhabit leaf litter and are less commonly found in the foliage or on tree trunks (Jäger et al. 2015). Most of these species are small-range endemics restricted to single mountains or forests (Zhang et al. 2023). Although the genus has been extensively studied, many new species are likely still undiscovered (Cao et al. 2016; Jiang et al. 2018; Zhang et al. 2023).

While examining Sparassidae collected from Yunnan and Jiangxi Provinces of China, as well as from Vinh Phuc Province of Vietnam, four new *Pseudopoda* species were identified. This study aims to provide diagnoses, morphological descriptions and photographic illustrations of these four new species.

## ﻿Material and methods

The specimens examined in this study were preserved in absolute ethanol and deposited at the Center for Behavioural Ecology and Evolution (CBEE), College of Life Sciences, Hubei University, Wuhan and at Xingtai University (XTU), Xingtai. Specimens were examined using an Olympus SZX7 stereo microscope. Photographs were taken using a Leica M205C stereo microscope. The male palp was examined and photographed after dissection. The epigyne was examined after being cleared with Proteinase K. Eye diameters were taken at the widest point. Legs and palp measurements are given as: total length (femur, patella, tibia, metatarsus [absent in palp], tarsus). All measurements are in millimetres (mm). Spination follows Davies (1994). The terminologies used in text and figure legends follow Quan et al. (2014).

Abbreviations in text: **AB**, anterior bands; **ALE**, anterior lateral eyes; **AME**, anterior median eyes; **C**, conductor; **CA**, carapace; **CH**, clypeus height; **CO**, copulatory opening; **dRTA**, dorsal retrolateral tibial apophysis; **E**, embolus; **EF**, epigynal field; **EP**, embolic projection; **FD**, fertilization duct; **Fe**, femur; **FW**, first winding; **IDS**, internal duct system; **LL**, lateral lobes; **Mt**, metatarsus; **OS**, opisthosoma; **Pa**, patella; **PLE**, posterior lateral eyes; **PME**, posterior median eyes; **Pp**, palp; **RTA**, retrolateral tibial apophysis; **S**, spermathecae; **Sp**, spermophor; **ST**, subtegulum; **T**, tegulum; **Ti**, tibia; **vRTA**, ventral retrolateral tibial apophysis; **I, II, III, IV**, legs I to IV.

## ﻿Taxonomy

**Family Sparassidae Bertkau, 1872**

**Subfamily Heteropodinae Thorell, 1873**

### ﻿Genus Pseudopoda

Taxon classificationAnimaliaAraneaeSparassidae

Jäger, 2000

E09638B3-71FE-508E-9535-15CCB1453D95

#### Type species.

*Pseudopodaprompta* (O. Pickard-Cambridge, 1885).

#### Diagnosis.

See Zhang et al. (2023).

#### Distribution.

East, South and Southeast Asia.

### ﻿ Pseudopoda campylotropa

Taxon classificationAnimaliaAraneaeSparassidae

Zhang, Chen, Liu, Jäger & Hu sp. nov.

712FFE63-E877-5126-B3A4-71E3A88F4337

https://zoobank.org/B043CE45-65A1-47E0-A081-A2A32A33F3FA

Figs 1 , 2 , 3 , 11

#### Type material.

***Holotype*** male: China, • Yunnan Province: Zhaotong City, Weixin County, Houshan Forest Farm, 27°51'01"N, 105°00'48"E, alt. 1637 m, 25 April 2024, Caifu Tao leg. (CBEE, LJ2024002). ***Paratypes***: • 3 males, 3 females, with same data as for holotype (CBEE, LJ2024003). 1 female, with same data as for holotype, except: 28 June 2020 (CBEE, LJ202005004).

#### Etymology.

The specific name is derived from Greek 'campylo' meaning 'bend, turn', and 'trop' derived from 'trepein' meaning 'to turn', referring to the crook-shaped distal part of E in ventral view; adjective.

#### Diagnosis.

**Figure 1.**
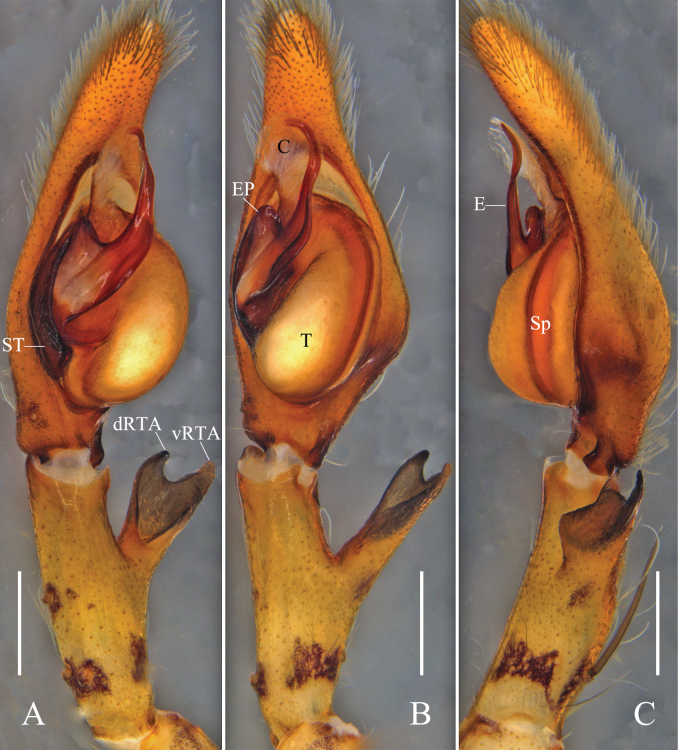
*Pseudopodacampylotropa* Zhang, Chen, Liu, Jäger & Hu, sp. nov., left male palp **A** prolateral **B** ventral **C** retrolateral. Abbreviations: C, conductor; dRTA, dorsal retrolateral tibial apophysis; E, embolus; EP, embolic projection; Sp, spermophor; ST, subtegulum; T, tegulum; vRTA, ventral retrolateral tibial apophysis. Scale bars: 0.5 mm.

**Figure 2.**
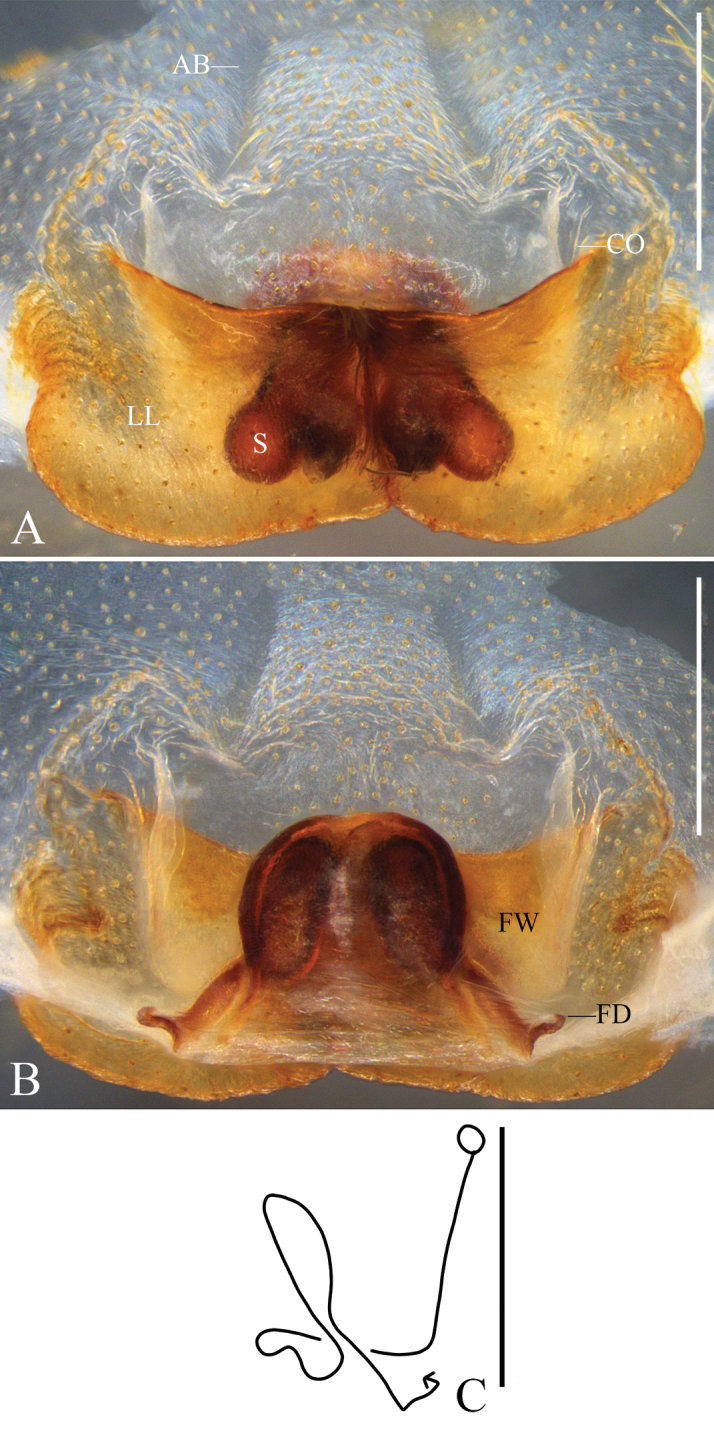
*Pseudopodacampylotropa* Zhang, Chen, Liu, Jäger & Hu, sp. nov., female **A** epigyne, ventral **B** vulva, dorsal **C** schematic course of internal duct system, dorsal. Abbreviations: AB, anterior bands; CO, copulatory opening; FD, fertilization duct; FW, first winding; LL, lateral lobes; S, spermathecae. Scale bars: 0.5 mm.

Males of *P.campylotropa* Zhang, Chen, Liu, Jäger & Hu, sp. nov. resemble those of *P.explanata* Zhang, Jäger & Liu, 2023 (cf. figs 1A–C vs. figs 103A–C in Zhang et al. 2023) by having a sickle-shaped E, a broad, distad EP, and a medially arising RTA with broad elongated base and distal tips of dRTA and vRTA, but can be recognised by: 1) EP short and triangular with short and rounded tip, and 2) dRTA with rounded tip (vs. EP long and trapezoidal, dRTA with thin and acute tip in *P.explanata*). Females of *P.campylotropa* Zhang, Chen, Liu, Jäger & Hu, sp. nov. resemble those of *P.curva* Zhang, Jäger & Liu, 2023 and *P.shimenensis* Zhang, Jäger & Liu, 2023 (cf. figs 2A–C vs. figs 76A–C and 219A–C in Zhang et al. 2023) by having the epigyne distinctly wider than long with the anterior and posterior margins of LL almost straight, but can be distinguished from both species by: IDS with short sclerotized tubes with spherical end in ventral view (vs. IDS with long and curved tubes with rounded, but not spherical end in *P.curva* and *P.shimenensis*).

#### Description.

**Male (holotype)**: Measurements: Small-sized. Body length 7.8, CA length 4.0, width 3.8; OS length 3.7, width 2.2. Eyes: AME 0.20, ALE 0.30, PME 0.24, PLE 0.25, AME–AME 0.18, AME–ALE 0.01, PME–PME 0.26, PME–PLE 0.32, AME–PME 0.24, ALE–PLE 0.27, CHAME 0.19, CHALE 0.23. Spination: Pp 131, 101, 3110; Fe I 312, II 313, III 322, IV 331; Pa I–II 101, IV 000; Ti I 1218, II 121(10), III 2124, IV 2126; Mt I 2024, II 2026, III 2124, IV 3036. Measurements of palp and legs: Pp 6.1 (2.0, 0.6, 1.3, –, 2.2), I 23.4 (6.2, 1.7, 7.0, 6.3, 2.2), II 25.3 (6.7, 1.8, 7.4, 7.1, 2.3), III 18.7 (5.2, 1.4, 5.3, 5.2, 1.6), IV 21.0 (5.9, 1.5, 5.5, 6.2, 1.9). Leg formula: II-I-IV-III. Chelicerae with 3 promarginal, 4 retromarginal teeth, and c. 26 intermarginal denticles.

Palp (Figs 1A–C): as in diagnosis. C membranous, arising from T at 11:30 o’clock position; prolateral margin of C slightly sclerotized in basal half. E slender and sickle-shaped in apical half, arising from 9 o’clock position of T. RTA arising medially from Ti; vRTA and dRTA with rounded tip.

**Figure 3.**
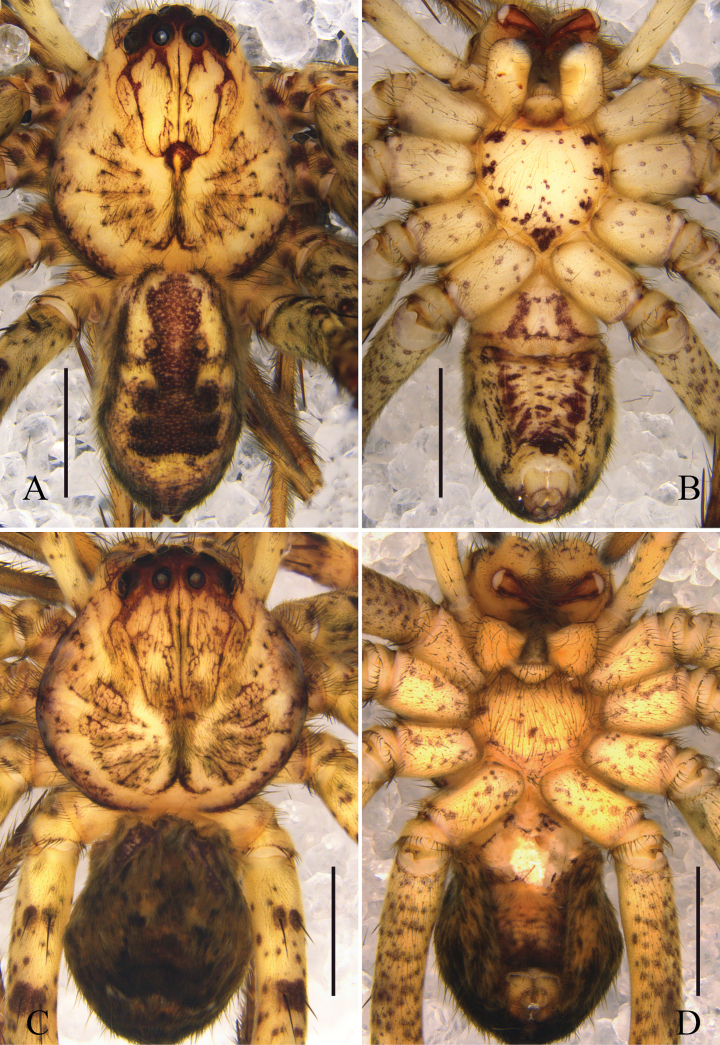
Habitus of *Pseudopodacampylotropa* Zhang, Chen, Liu, Jäger & Hu, sp. nov. **A, B** male (**A** dorsal **B** ventral) **C, D** female (**C** dorsal **D** ventral). Scale bars: 2 mm.

Colouration (Figs 3A, B): CA yellow, with dark reddish-brown spots and lines, eye area reddish-brown, posterior margins dark brown. Fovea dark brown. Sternum and legs yellow, with dark reddish-brown spots. OS yellow, dorsum with distinct dark reddish-brown pattern of elongated heart-patch and three transversal fused chevrons, venter with some reddish-brown spots, some of them fused.

**Female (paratype)**: Measurements: Small-sized. Body length 7.7, CA length 4.0, width 4.2; OS length 3.7, width 2.9. Eyes: AME 0.19, ALE 0.30, PME 0.27, PLE 0.24, AME–AME 0.18, AME–ALE 0.06, PME–PME 0.25, PME–PLE 0.32, AME–PME 0.26, ALE–PLE 0.28, CHAME 0.35, CHALE 0.32. Spination: Pp 121, 101, 3130, 3020; Fe I 313, II 213, III 312, IV 311; Pa I–IV 101; Ti I 2227, II 111(10), III 2116, IV 2025; Mt I–II 2024, III 2124, IV 3034. Measurements of palp and legs: Pp 5.3 (1.6, 0.7, 1.3, –, 1.7), I 15.1 (4.4, 1.5, 4.2, 3.6, 1.4), II 16.4 (4.9, 1.5, 4.6, 3.9, 1.5), III 12.1 (3.7, 1.3, 3.3, 2.7, 1.1), IV 14.1 (4.4, 1.4, 3.5, 3.4, 1.4). Leg formula: II-I-IV-III. Chelicerae with 3 promarginal, 4 retromarginal teeth, and c. 26 intermarginal denticles.

Epigyne (Figs 2A–C): as in diagnosis. EF wider than long, AB indistinct. LL touching each other along the middle lines with a slight asymmetry, i.e. right LL larger than left one. Anterior margins of LL almost parallel to posterior part. FW membranous, with straight lateral margins. IDS with round cover of spermathecae, the latter sub-parallel and elongate. FD long and narrow, posteriorly diverging.

Colouration (Figs 3C, D): as in male, but OS generally darker.

#### Distribution.

Known only from the type locality (Fig. 11).

#### Remarks.

This species shares the same collecting locality with *P.taoi* Zhang, Jäger & Liu, 2023, but exhibits distinct differences in coloration and pattern (e.g., yellow habitus vs. reddish-brown habitus, and CA, sternum and legs with dark reddish-brown spots vs. without spots), which led us to consider them as different species. The colouration and patterns on the male and female habitus of *P.campylotropa* Zhang, Chen, Liu, Jäger & Hu, sp. nov. show strong support for our matching (cf. figs 3A–D vs. figs 241A, B in Zhang et al. 2023). Further research and collection of material of both sexes are needed to verify this taxonomic decision.

### ﻿ Pseudopoda caoguii

Taxon classificationAnimaliaAraneaeSparassidae

Zhang, Chen, Liu, Jäger & Hu sp. nov.

D5790213-0F6E-5ACE-9ED8-D1E68135D312

https://zoobank.org/47CC428F-E716-4D75-B3BE-E873DB46C27B

Figs 4 , 5 , 11

#### Type material.

***Holotype*** female: China, • Yunnan Province: Zhaotong City, Weixin County, Daxueshan Forest Farm, 27°52'48"N, 104°46'29"E, alt. 1560 m, 21 July 2024, Gui Cao leg. (CBEE, LJ2024001).

#### Etymology.

The specific name is derived from the name of the collector; noun in genitive case.

#### Diagnosis.

**Figure 4.**
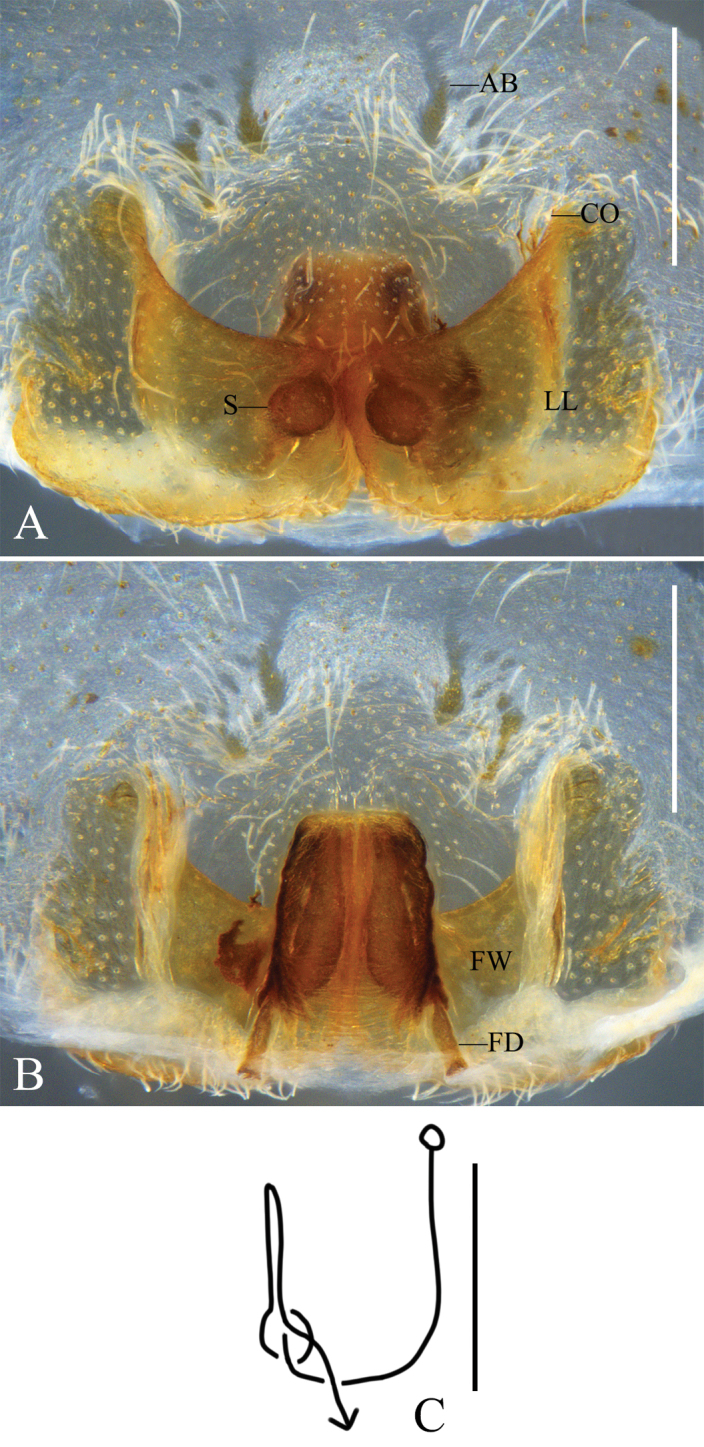
*Pseudopodacaoguii* Zhang, Chen, Liu, Jäger & Hu, sp. nov., female **A** epigyne, ventral **B** vulva, dorsal **C** schematic course of internal duct system, dorsal. Abbreviations: AB, anterior bands; CO, copulatory opening; FD, fertilization duct; FW, first winding; LL, lateral lobes; S, spermathecae. Scale bars: 0.5 mm.

Females of *P.caoguii* Zhang, Chen, Liu, Jäger & Hu, sp. nov. resemble those of *P.langyaensis* Zhang, Jäger & Liu, 2023 (cf. figs 4A–C vs. figs 140A–C in Zhang et al. 2023) by having wide U-shaped anterior margins of LL and spherical S, but can be distinguished by: 1) the posterior margins of LL almost straight, 2) body colouration light yellowish-brown (vs. posterior margins of LL distinctly lobed and body colouration reddish-brown in *P.langyaensis*).

#### Description.

**Female (holotype)**: Measurements: Medium-sized. Body length 12.6, CA length 4.8, width 4.3; OS length 7.3, width 4.1. Eyes: AME 0.18, ALE 0.22, PME 0.19, PLE 0.29, AME–AME 0.22, AME–ALE 0.14, PME–PME 0.32, PME–PLE 0.34, AME–PME 0.21, ALE–PLE 0.28, CHAME 0.36, CHALE 0.35. Spination: Pp 121, 101, 3130, 3030; Fe I 313, II 323, III 322, IV 321; Pa I–II 101, III 001, IV 000; Ti I–II 2226, III–IV 2126; Mt I–II 2024, III 2124, IV 3036. Measurements of palp and legs: Pp 7.2 (2.2, 1.0, 1.5, –, 2.5), I 22.9 (6.5, 1.8, 6.6, 5.8, 2.2), II 24.9 (7.2, 2.0, 7.1, 6.3, 2.3), III 17.6 (5.5, 1.8, 4.7, 4.1, 1.5), IV 19.5 (6.2, 1.5, 5.0, 4.9, 1.9). Leg formula: II-I-IV-III. Chelicerae with 3 promarginal, 4 retromarginal teeth, and c. 25 intermarginal denticles.

Epigyne (Figs 4A–C): as in diagnosis. EF wider than long, with distinct AB. LL touching each other along median line, anterior margins of LL forming a wide “U”. FW membranous, with straight lateral margins, covering whole IDS. FD short and narrow.

**Figure 5.**
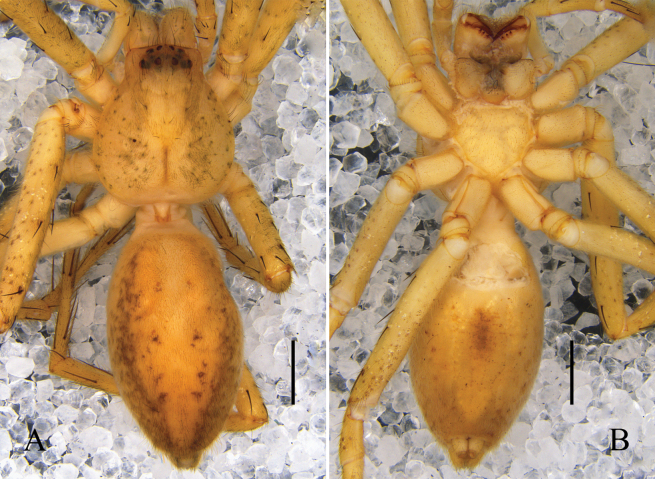
Habitus of female *Pseudopodacaoguii* Zhang, Chen, Liu, Jäger & Hu, sp. nov. **A** dorsal **B** ventral. Scale bars: 2 mm.

Colouration (Figs 5A, B): CA yellow, with black spots, median band of CA lighter than rest, fovea reddish-brown. Sternum light yellow. Legs yellow, with black spots. OS dorsally orange, with black marks, median band of OS lighter than rest; ventrally with a reddish-brown patch medially.

**Male**: Unknown.

#### Distribution.

Known only from the type locality (Fig. 11).

#### Remarks.

This species might potentially be conspecific with *P.taoi* Zhang, Jäger & Liu, 2023, given that the two localities are only about 20 kilometres apart. However, there are distinct somatic differences in the female compared to the male of *P.taoi*, including: 1) yellow to orange habitus vs. reddish-brown habitus, 2) OS dorsally with several dark regularly arranged spots vs. OS dorsally dark brown with some light spots, 3) OS ventrally with a reddish-brown patch in middle part behind epigastric furrow vs. OS ventrally without such distinct patch, 4) prosoma dorsally with distinct light median band without dark pattern inside and only few dark dots in lateral parts vs. median band with distinct dark pattern anterior, along and posterior of fovea and distinct dark pattern, partly fused in lateral parts (cf. figs 5A, B vs. figs 241A, B in Zhang et al. 2023). These differences indicate that the present material likely represents a different species than *P.taoi*. Moreover, *Pseudopoda* species have a very similar colouration pattern in both sexes and colour dimorphisms are almost absent. Further research and future findings are needed to resolve this ambiguity conclusively.

### ﻿ Pseudopoda yejiachangensis

Taxon classificationAnimaliaAraneaeSparassidae

Zhang, Chen, Liu, Jäger & Hu sp. nov.

537B70AB-1F16-558A-B7D5-C5AF79337BCB

https://zoobank.org/596F96F8-D9AB-4377-9B88-2864A8EAFA37

Figs 6 , 7 , 11

#### Type material.

***Holotype*** male: China, • Jiangxi Province: Shangrao City, Yanshan County, Jiangxi Wuyi Mountain National Nature Reserve, Yejiachang, 27°50'37"N, 117°44'00"E, alt. 889 m, 13 September 2024, Chenliang Li & Wanyu Li leg. (XTU, INS-R001).

#### Etymology.

The specific name is derived from the type locality; adjective.

#### Diagnosis.

**Figure 6.**
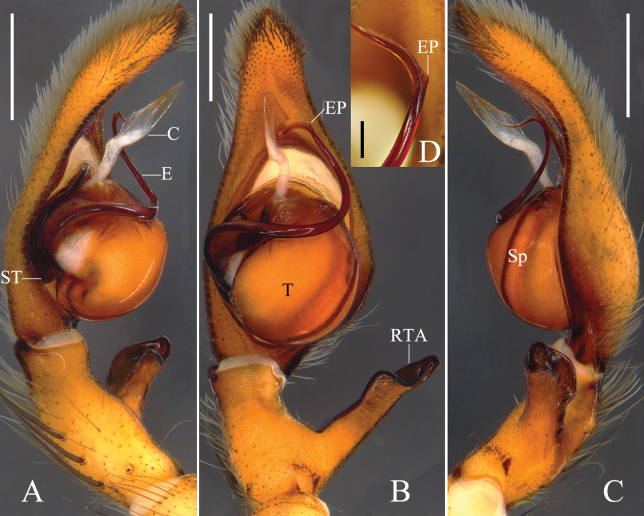
*Pseudopodayejiachangensis* Zhang, Chen, Liu, Jäger & Hu, sp. nov., left male palp **A** prolateral **B** ventral **C** retrolateral **D** detail of EP. Abbreviations: C, conductor; E, embolus; EP, embolic projection; RTA, retrolateral tibial apophysis; Sp, spermophor; ST, subtegulum; T, tegulum. Scale bars: 0.5 mm (**A–C**); 0.1 mm (**D**).

Males of *P.yejiachangensis* Zhang, Chen, Liu, Jäger & Hu, sp. nov. resemble those of *P.shuqiangi* Jäger & Vedel, 2007, *P.lushanensis* (Wang, 1990), and *P.jiugongensis* Zhang, Jäger & Liu, 2023 (cf. figs 6A–D vs. figs 73–76 in Jäger and Vedel 2007, figs 4A–C, 5A–C in Quan et al. 2014, and figs 132A–C in Zhang et al. 2023) by having a similar long filiform E and similar simple RTA, but can be recognised from *P.shuqiangi* by: 1) subterminal E with a tiny tooth-shaped EP, and 2) width of RTA obviously thinner than Ti in venter view (vs. E without EP, width of RTA almost equal to Ti in *P.shuqiangi*); it can be recognised from *P.lushanensis* by: 1) E thin throughout its entire length, except embolic base, 2) subterminal E with a tiny tooth-shaped EP, and 3) RTA broad throughout its entire length, arising from Ti proximally (vs. both basal and proximal E broad, EP absent, and RTA finger-shaped, arising subdistally from Ti in *P.lushanensis*); it can be recognised from *P.jiugongensis* by: 1) E arising from T at 9 o’clock position, 2) subterminal E with a tiny tooth-shaped EP, and 3) RTA broad throughout its entire length, arising from Ti proximally (vs. E arising from T at 10:30 o’clock position, EP absent, and RTA finger-shaped with a blunt tip, arising medially from Ti in *P.jiugongensis*).

#### Description.

**Male (holotype)**: Measurements: Small-sized. Body length 8.3, CA length 3.7, width 3.6; OS length 4.5, width 2.7. Eyes: AME 0.17, ALE 0.29, PME 0.20, PLE 0.27, AME–AME 0.13, AME–ALE 0.06, PME–PME 0.25, PME–PLE 0.32, AME–PME 0.26, ALE–PLE 0.23, CHAME 0.31, CHALE 0.30. Spination: Pp 131, 101, 3000; Fe I 523, II 323, III 322, IV 331; Pa I–II 001, III–IV 000; Ti I 1118, II 1116, III–IV 2126; Mt I–IV 2024. Measurements of palp and legs: Pp 5.7 (1.9, 0.8, 0.9, –, 2.1), I 18.1 (4.8, 1.3, 5.4, 4.9, 1.7), II 19.7 (5.7, 1.4, 5.7, 5.2, 1.7), III 14.9 (4.6, 1.2, 4.1, 3.8, 1.2), IV 17.2 (5.3, 1.1, 4.7, 4.6, 1.5). Leg formula: II-I-IV-III. Chelicerae with 3 promarginal, 4 retromarginal teeth, and c. 33 intermarginal denticles.

Palp (Figs 6A–D): as in diagnosis. T spherical. C long and membranous, arising from T in 12 o’clock position. E long and filiform, arising in 9 o’clock position from T; E running retrolaterally first, then distally, finally ventrally to distally; subterminal E with a tiny tooth-shaped EP. Length of RTA almost same as Ti.

**Figure 7.**
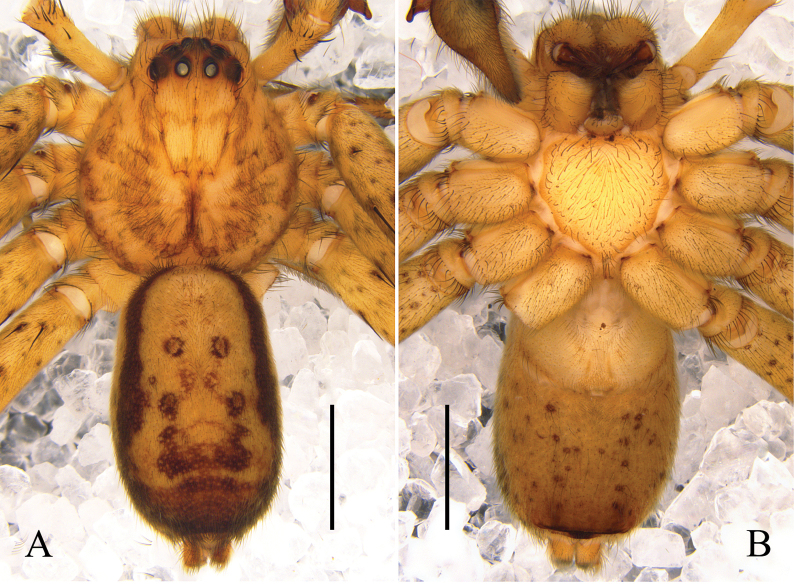
Habitus of male *Pseudopodayejiachangensis* Zhang, Chen, Liu, Jäger & Hu, sp. nov. **A** dorsal **B** ventral. Scale bars: 2 mm.

Colouration (Figs 7A, B): CA yellow, with brown spots and dense cover of setae, both latter forming two lateral bands. Fovea dark. Sternum yellow. Legs yellow, with brown spots and spine patches. OS yellow, dorsum with dark brown margin and brown patches pairwise arranged, posteriorly fused, venter with some small brown spots and dark transversal patch posteriorly.

**Female**: Unknown.

#### Distribution.

Known only from the type locality (Fig. 11).

#### Remarks.

This species was collected from the same mountain range as *P.papilionacea* Zhang, Jäger & Liu, 2023. However, both sexes of *P.papilionacea* are known (Zhang et al. 2023). In addition, the nearest species of the genus, *P.longxiensis* Zhang, Jäger & Liu, 2023, is distributed about 150 km from this species (outside the endemic range of most *Pseudopoda* species, personal observation), and the distinct differences of habitus (e.g., CA with brown patterns and dense cover of setae vs. CA with black spots, without setae) (cf. figs 7A, B vs. figs 156A, B in Zhang et al. 2023) indicating that they are likely not conspecific. Further research and future findings are needed to resolve this ambiguity conclusively.

### ﻿ Pseudopoda ornithorhynchus

Taxon classificationAnimaliaAraneaeSparassidae

Zhang, Chen, Liu, Jäger & Hu sp. nov.

26D8DA06-56A8-55D4-8585-AD05C62B8CB3

https://zoobank.org/9311A7A5-5244-431F-823E-DD90B3F4D1E5

**Figure 8.**
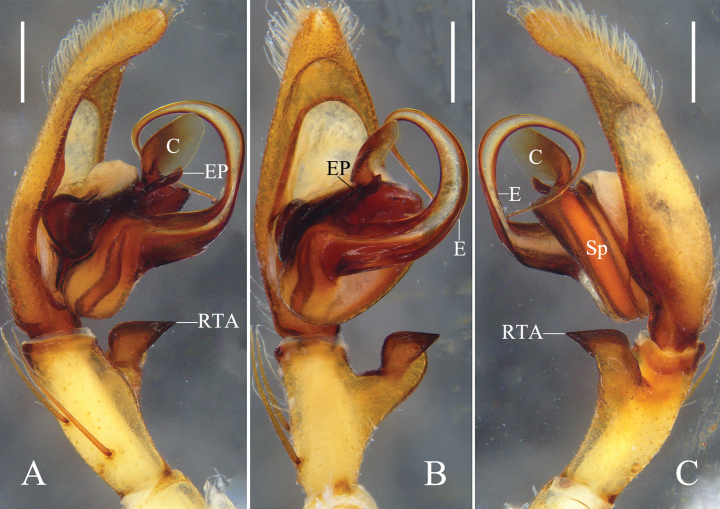
*Pseudopodaornithorhynchus* Zhang, Chen, Liu, Jäger & Hu, sp. nov., left male palp **A** prolateral **B** ventral **C** retrolateral. Abbreviations: C, conductor; E, embolus; EP, embolic projection; RTA, retrolateral tibial apophysis; Sp, spermophor. Scale bars: 0.5 mm.

**Figure 9.**
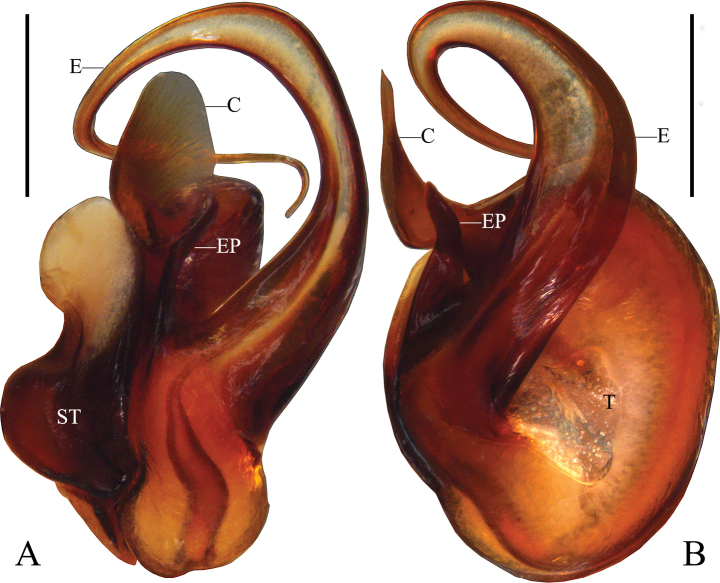
*Pseudopodaornithorhynchus* Zhang, Chen, Liu, Jäger & Hu, sp. nov., left male palpal bulb **A** prolateral **B** ventral. Abbreviations: C, conductor; E, embolus; EP, embolic projection; ST, subtegulum; T, tegulum. Scale bars: 0.5 mm.

**Figure 10.**
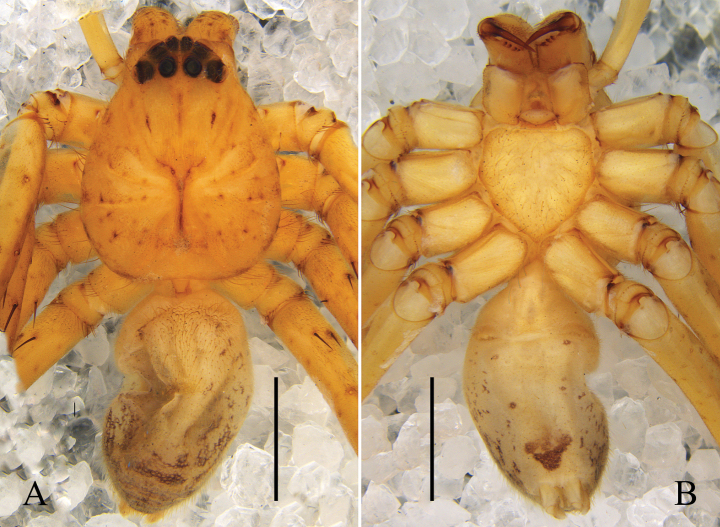
Habitus of male *Pseudopodaornithorhynchus* Zhang, Chen, Liu, Jäger & Hu, sp. nov. **A** dorsal **B** ventral. Scale bars: 2 mm.

**Figure 11.**
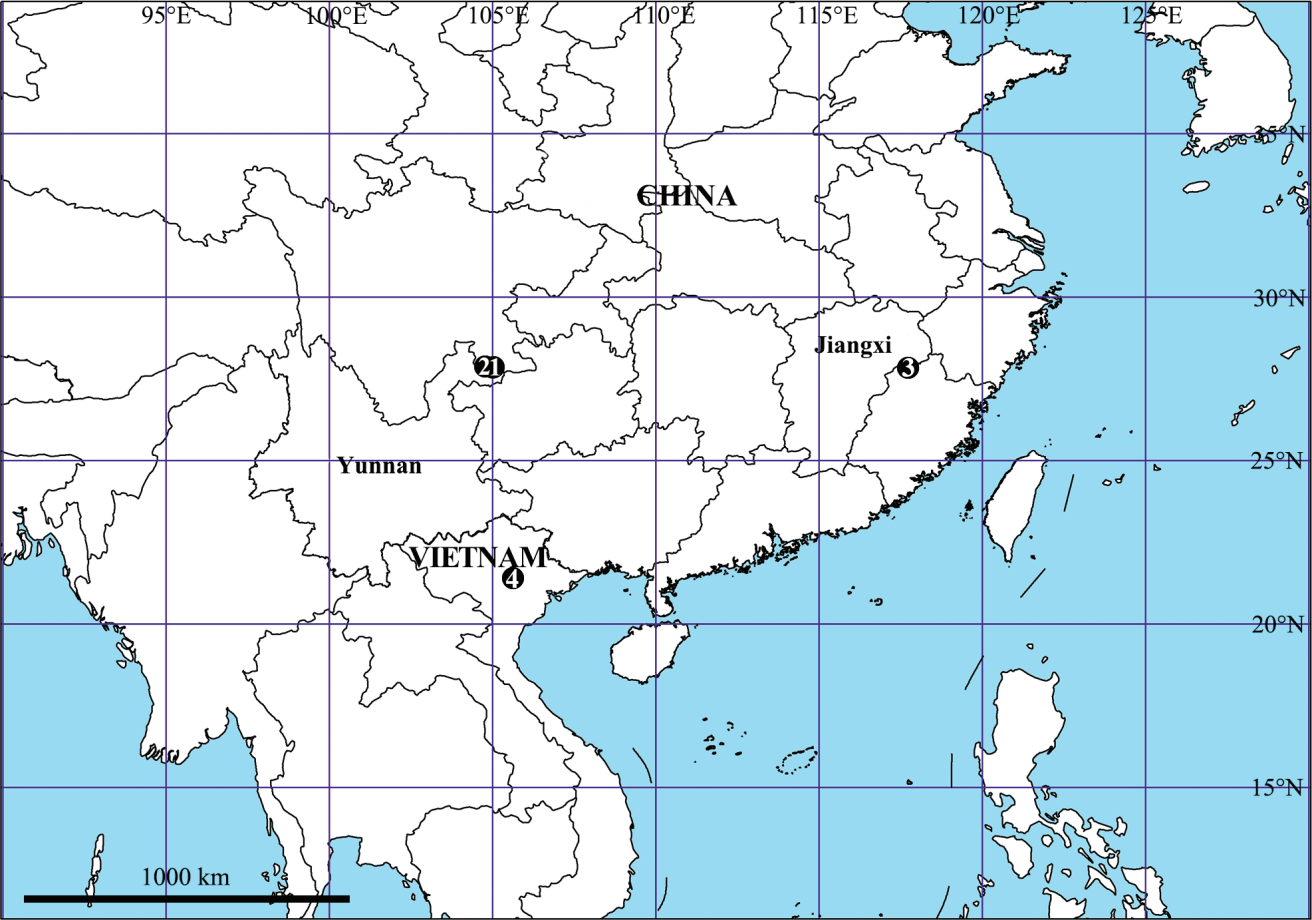
Distribution map of the four new *Pseudopoda* species **1***P.campylotropa* Zhang, Chen, Liu, Jäger & Hu, sp. nov. **2***P.caoguii* Zhang, Chen, Liu, Jäger & Hu, sp. nov. **3***P.yejiachangensis* Zhang, Chen, Liu, Jäger & Hu, sp. nov. **4***P.ornithorhynchus* Zhang, Chen, Liu, Jäger & Hu, sp. nov.

Figs 8 , 9 , 10 , 11

#### Type material.

***Holotype*** male: Vietnam: • Vinh Phuc Province: Tam Dao National Park, Pitfall trap, 21°25'7"N, 105°37'24"E, alt. 80 m, 1–30 December 2007, Dinh Sac Pham leg. (CBEE, LJ201804699).

#### Etymology.

The specific name is derived from Greek 'ornitho' meaning 'bird', and 'rhynchus' meaning 'beak', referring to the shape of the RTA; adjective.

#### Diagnosis.

Males of *P.ornithorhynchus* Zhang, Chen, Liu, Jäger & Hu, sp. nov. resemble those of *P.yangtaiensis* Zhang, Jäger & Liu, 2023 (cf. figs 8A–C, 9A, B vs. figs 268A–C in Zhang et al. 2023) by having a bird’s beak-shaped RTA, but can be recognised by: 1) E without basal protrusion, 2) EP short and digitiform, and 3) basal RTA columnar (vs. E with a basal prolateral protrusion, EP long and lamellar, arising from T at 1 o’clock position, basal RTA lamellar in *P.yangtaiensis*).

#### Description.

**Male (holotype)**: Measurements: Small-sized. Body length 7.6, CA length 3.8, width 3.2; OS length 3.6, width 2.3. Eyes: AME 0.15, ALE 0.29, PME 0.29, PLE 0.29, AME–AME 0.11, AME–ALE 0.04, PME–PME 0.12, PME–PLE 0.31, AME–PME 0.24, ALE–PLE 0.19, CHAME 0.43, CHALE 0.32. Spination: Pp 131, 101, 3100; Fe I 323, II 313, III 323, IV 331; Pa I–II 101, III 001, IV 000; Ti I 2128, II 1116, III–IV 2126; Mt I–III 2024, IV 3036. Measurements of palp and legs: Pp 6.3 (2.0, 0.8, 1.1, –, 2.4), I 22.8 (6.2, 1.6, 6.7, 6.0, 2.3), II 23.6 (6.2, 1.5, 7.2, 6.4, 2.3), III 17.7 (5.2, 1.3, 5.0, 4.5, 1.7), IV 21.9 (6.3, 1.4, 5.7, 6.4, 2.1). Leg formula: II-I-IV-III. Chelicerae with 3 promarginal, 4 retromarginal teeth, and c. 28 intermarginal denticles.

Palp (Figs 8A–C, 9A, B): as in diagnosis. C slightly sclerotized throughout, arising from T at 11 o’clock position. E strongly elongated with one distal coil, with broad basal part and thin terminal part, arising from 8:30 o’clock position of T. EP digitiform. RTA arising medially from Ti, tip retrolaterad.

Colouration (Figs 10A, B): CA yellowish-brown with few dots. Fovea dark reddish-brown. Sternum and legs yellow, the latter with faint dots. OS yellow, dorsum with irregular brown markings, especially in the posterior half, i.e. transversal bars, venter with brown spots, with an inverted triangle brown marking anterior to spinnerets.

**Female**: Unknown.

#### Distribution.

Known only from the type locality (Fig. 11).

#### Remarks.

This species is located about 140 km from *P.anfracta* Zhang, Jäger & Liu, 2023 and *P.zhengi* Zhang, Jäger & Liu, 2023 (outside the endemic range of most *Pseudopoda* species; personal observation, Zhang et al. 2023), and the unique character and colouration of the habitus (eye area as wide as head region, generally yellowish-brown in *P.ornithorhynchus* Zhang, Chen, Liu, Jäger & Hu, sp. nov. vs. the width of eye area almost two-thirds of head region, generally reddish-brown in the latter two known species) (cf. figs 10A, B vs. figs 13A, B, 280A, B in Zhang et al. 2023) indicating that with the specimen described here is likely not conspecific with either *P.anfracta* or *P.zhengi*. Further research and future findings are needed to resolve this ambiguity conclusively.

## Supplementary Material

XML Treatment for Pseudopoda

XML Treatment for Pseudopoda campylotropa

XML Treatment for Pseudopoda caoguii

XML Treatment for Pseudopoda yejiachangensis

XML Treatment for Pseudopoda ornithorhynchus

## ﻿Additional information

### Author ORCIDs

He Zhang https://orcid.org/0000-0002-1478-9837

Hailun Chen https://orcid.org/0009-0009-0879-6887

Jie Liu https://orcid.org/0000-0001-7744-9744

Peter Jäger https://orcid.org/0000-0003-1563-0147

Changhao Hu https://orcid.org/0009-0007-5591-3121

### Data availability

All of the data that support the findings of this study are available in the main text.
